# Neural Network Detection of Pacemakers for MRI Safety

**DOI:** 10.1007/s10278-022-00663-2

**Published:** 2022-06-29

**Authors:** Mark Daniel Vernon Thurston, Daniel H Kim, Huub K Wit

**Affiliations:** 1 Peninsula Medical School, University of Plymouth, Plymouth Science Park, Plymouth, PL6 8BT UK; 2 The Department of Clinical Imaging, The Royal Cornwall Hospitals NHS Trust, Truro, UK; 3 The Radiology Department, Torbay and South Devon NHS Trust, Torquay, UK; 4 University Hospitals Plymouth NHS Trust, Plymouth, Devon PL6 8DH UK

**Keywords:** Artificial intelligence, Cardiac devices, Image classification, MRI, Patient safety

## Abstract

Flagging the presence of cardiac devices such as pacemakers before an MRI scan is essential to allow appropriate safety checks. We assess the accuracy with which a machine learning model can classify the presence or absence of a pacemaker on pre-existing chest radiographs. A total of 7973 chest radiographs were collected, 3996 with pacemakers visible and 3977 without. Images were identified from information available on the radiology information system (RIS) and correlated with report text. Manual review of images by two board certified radiologists was performed to ensure correct labeling. The data set was divided into training, validation, and a hold-back test set. The data were used to retrain a pre-trained image classification neural network. Final model performance was assessed on the test set. Accuracy of 99.67% on the test set was achieved. Re-testing the final model on the full training and validation data revealed a few additional misclassified examples which are further analyzed. Neural network image classification could be used to screen for the presence of cardiac devices, in addition to current safety processes, providing notification of device presence in advance of safety questionnaires. Computational power to run the model is low. Further work on misclassified examples could improve accuracy on edge cases. The focus of many healthcare applications of computer vision techniques has been for diagnosis and guiding management. This work illustrates an application of computer vision image classification to enhance current processes and improve patient safety.

## Introduction

Screening of patients for pacemakers and other cardiac devices prior to magnetic resonance imaging (MRI) is vital to ensure the patient and device can be scanned safely. Most modern pacemakers are categorized as “MR-conditional.” For these implants, MRI is not absolutely contraindicated but the device needs careful prior assessment to ensure the scan takes place under manufacturer-specified conditions. Safe examination requires review of medical records and co-ordination of multiple experts [1]: for example, a post-scan device check by a cardiac technician is usually needed to ensure continued optimal and safe function [2, 3]. Late detection has the potential to result in last minute cancellations and wasted scanner time, if a cardiac technician is not available for the post-scan device check. Failure to perform the required checks can result in device dysfunction with potential harm to the patient.

Absence of ionizing radiation, excellent tissue characterization, and high spatial resolution make MRI the standard imaging modality for many cardiac and non-cardiac conditions [4]. One estimate suggested that between 50 and 75% of patients with cardiac devices may require an MRI scan during their lifetime [5]. Appropriate screening policies and procedures are therefore essential before permitting entry to the MRI scanner to prevent injury [6]. Best practice is to use referrer and patient questionnaires to identify patients with devices (or other issues) that need further investigation. Questionnaires are not fail-safe as referrer responses can be unreliable and patient responses are often not available until the day of the scan.

In the age of digital picture archiving and communication systems (PACS), a significant majority of patients with cardiac disease (and the subgroup of these with pacemakers) will have had a previous chest radiograph revealing the presence of the device. Human error in radiology is inevitable [7] but failure frequently offers rich learning opportunities [8].

Artificial intelligence has progressed exponentially since Alan Turing’s seminal 1950 definition as “can machines think?” [9]; François Chollet’s recent definition is more specific: “the effort to automate intellectual tasks normally performed by humans” [10]. Deep neural networks are a subset of artificial intelligence increasingly used in a broad range of applications. A subset, convolutional neural networks, is widely used for image classification tasks. Within healthcare, artificial intelligence techniques have been applied to a diverse range of applications including molecular imaging assessment [11], fracture recognition [12], plain radiograph analysis [13, 14], bone density scoring [15], and missed appointment attendances [16] to name just a few.

We describe the design of a neural network–based model for identification of the presence of pacemakers from chest radiographs with the aim of identifying the presence of these devices automatically. This has the potential to improve MRI safety and reduce last-minute cancellations.

## Materials and Methods

Two hospital sites (reflecting different patient populations) were included for improved model generalizability. Hospital 1 is a medium-sized (760 beds) teaching hospital and Hospital 2 is a large teaching hospital (1000 beds) with tertiary cardiology and cardiothoracic services. The study design was retrospective and observational using preexisting medical image data.

### Subject Inclusion

A database search was performed on the radiology information system (RIS) to identify any patient with a pacemaker insertion event. These patients were identified using the National Interim Clinical Imaging Procedures (NICIP) code IPACEI. From these patients, two separate groups were created for each of the 2 sites. The number of samples was chosen with the aim of providing adequate power whilst still allowing review by 2 radiologists. The date range of the database search included May 2006 to February 2020. The first 2000 chest radiograph examinations on a list matching the following criteria were selected:

1. All chest radiograph examinations taking place before the pacemaker insertion. To reduce false positives, those with “pacemaker” mentioned in the report were excluded.
2. All chest radiographs examinations taking place after the pacemaker insertion event. To reduce false negatives, only those with “pacemaker” mentioned in the radiology report were included.

This technique was chosen to select similar subjects in both populations: paced and unpaced examples coming from the same patient group (pre- and post- device insertion).

Although simple and effective, a weakness of this search methodology was that using the keyword “pacemaker” did not include other devices such as Automated Implantable Cardioverter Defibrillators (AICD).

### Image Data Acquisition

For each examination on the list, pixel data for each chest X-ray event were downloaded and saved with no patient identifying information. The image download pipeline was created using *bash* (for Hospital 2) and *PowerShell* (for Hospital 1) with *dcmtk* (OFFIS computer science institute) [17] performing the image download step. Pixel values were normalized; the window values were not adjusted. Anonymized pixel data were stored in labeled paced and unpaced categories for each participating site in portable network graphic (PNG) format. A cryptographic-grade one-way hash function (SHA-3) based on a unique study identifier was used to ensure that no duplicate studies were included whilst maintaining anonymity.

Data were collected with the following aims:

- 50:50 balance between sites
- 50:50 balanced split of paced and unpaced patients

The final set included less than 2000 images per category, as image download error resulted in failure of image storage for in a small number of cases.

### Ground Truth Confirmation

The database search technique returned a high rate of correctly categorized images. Accurate training set labels are critical for high model performance on unseen data. To ensure correct labeling, each image was reviewed by two board certified (FRCR) radiologists. Any discrepancies were discussed at a mediation meeting. Images where a human would be unable to categorize (even on close scrutiny) were removed from the final set (e.g., artifact distorting the entire image). Images that could be correctly classified, however difficult, were retained: for example, abandoned leads, pacing box on the edge of the film.

Image curation was performed before the model training. The majority of removed images were incorrectly classified paced chest X-rays in the unpaced group. Pacemaker insertion may have taken place either before the start of digital records or at another center.

Many lateral chest radiographs were unexpectedly included and these were more numerous within the paced image class. There were also several images in which the field of view only included the inferior part of the chest. If correctly labeled, these were left in the final data set, in compliance with the research protocol. In retrospect, revised inclusion criteria stating satisfactory diagnostic frontal chest X-rays (limiting to those including the full chest) would result in improved model accuracy with better generalizability (Table 1).

### Split

**Table 1:** Data set sizes

	**Paced**		**Unpaced**	
Hospital 1	1997	1991	1996	1939
Hospital 2	1999	1965	1981	1953
Total	3996	3956	3977	3892

The following randomly allocated subsets were created from the full curated data set:

- Model training:
  - 6039 (80%) training set
  - 1509 (20%) validation set
- Test set:
  - 300 examples (150 paced, 150 unpaced) kept back for assessment of the final model

### Neural Network Architecture

A Python-based deep neural network was built with *Keras* [18] using the *TensorFlow* [19] backend. Graphics processing unit (GPU) hardware acceleration was used for neural network training. *Jupyter Lab* [20] was used for model development to enable iterative improvements to be made efficiently.

A convolutional neural network based on a pre-trained model was selected as a proven choice for computer vision and image classification tasks using transfer learning. Several different pre-trained base networks were trialed, including *VGG16* [21] and *Inception V3* [22]. Following curve analysis for each model, *Inception V3* achieved the smallest loss on the validation set and was chosen for the final model.

Images were shuffled and resized to 299 × 299 to enable compatibility with the target neural network. After each adjustment of the hyperparameters, the performance on the validation set was used to assess the effect on model performance. Accuracy and loss graphs against the training and cross-validation sets were produced. These were inspected after each small adjustment to the model hyperparameters. Learning curves (with corresponding hyperparameters) for each iteration were kept for reference.

### Model Training

**Fig. 1:**
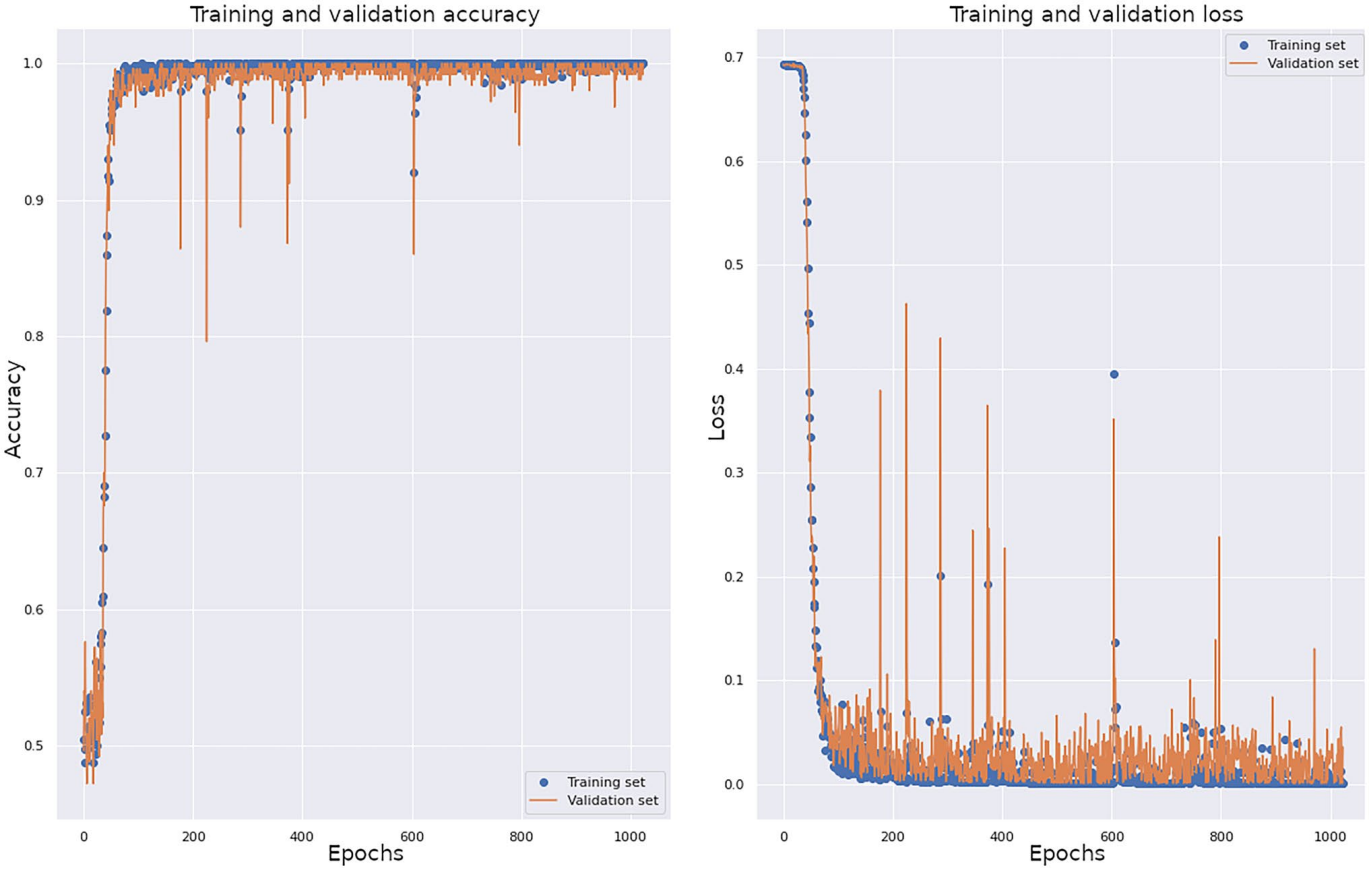
Accuracy and loss on the training and validation sets

The final model was trained for 1024 epochs using stochastic gradient descent (SGD) with Nestarov momentum. The binary cross-entropy loss function was utilized. The data set was augmented with horizontal flip (in case of pacemaker boxes sitting on either side of the chest). The model achieving the lowest loss on the validation set during training was saved using a checkpoint (Fig. 1).

## Results

**Fig. 2:**
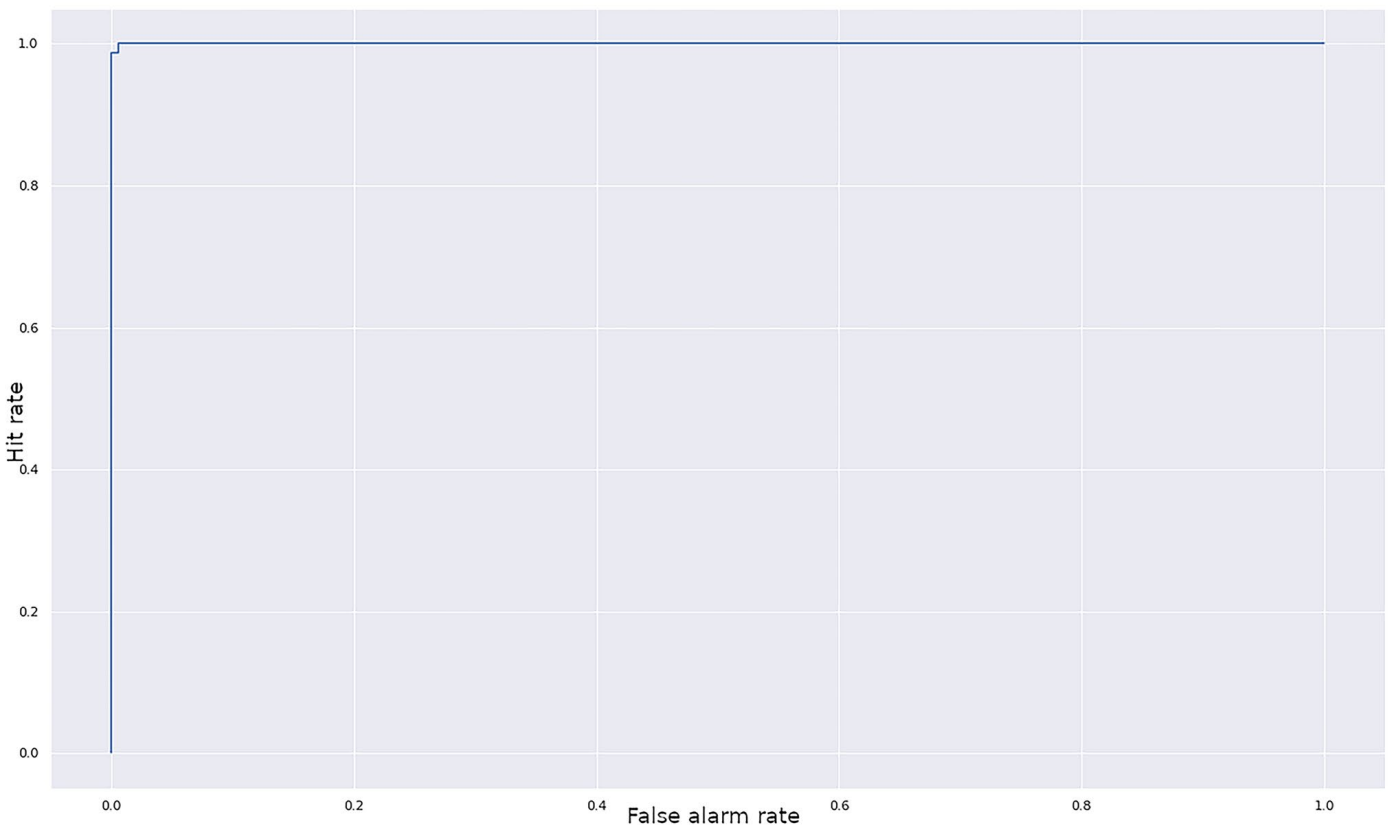
Receiver operating characteristic (ROC) curve

The final model achieved an accuracy of 99.67%, correctly classifying 299 out of the 300 test set images. Sensitivity on the test set was 100%; specificity 99.3% (Fig. 2).

### Incorrectly Classified Examples

**Fig. 3:**
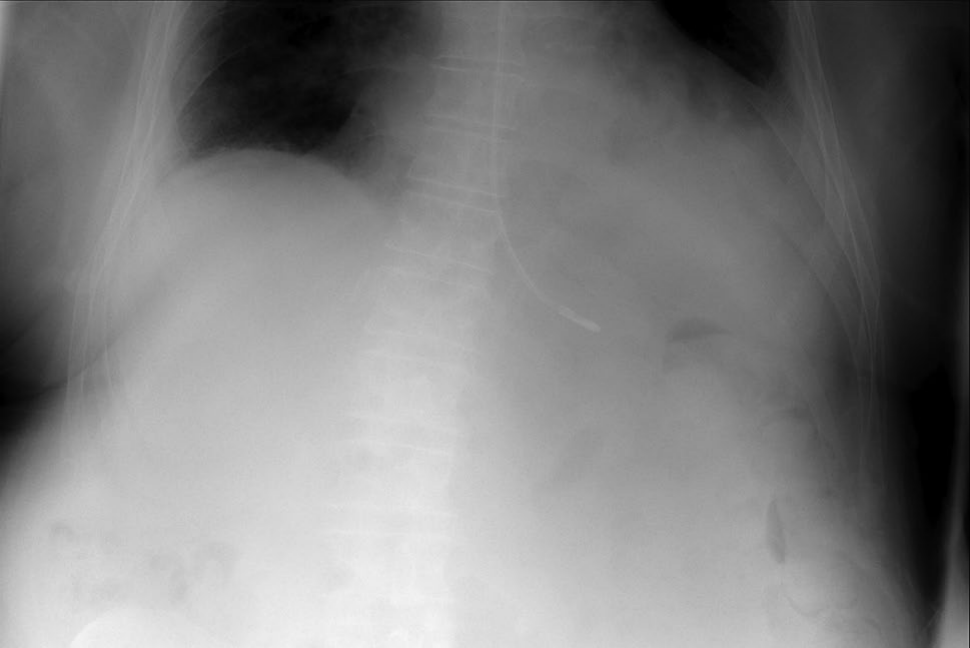
Incorrectly classified test set image. False positive classification as a nasogastric feeding tube has been incorrectly identified as a pacemaker lead

The single incorrectly classified image in the test set shows a feeding tube. The chest radiograph appearances are very similar to a pacemaker lead (Fig. 3)

### Full Data Set

The test data set, in retrospect, was relatively small given the high model performance. Given that only one incorrectly classified image was present in the test data set, the final model was run on the full data set to classify image. Analysis of incorrectly classified examples was performed to analyze patterns of error.

The authors acknowledge that running predictions on the training set is not best practice but this was carried out to allow further analysis which would not have otherwise been possible.

**Fig. 4:**
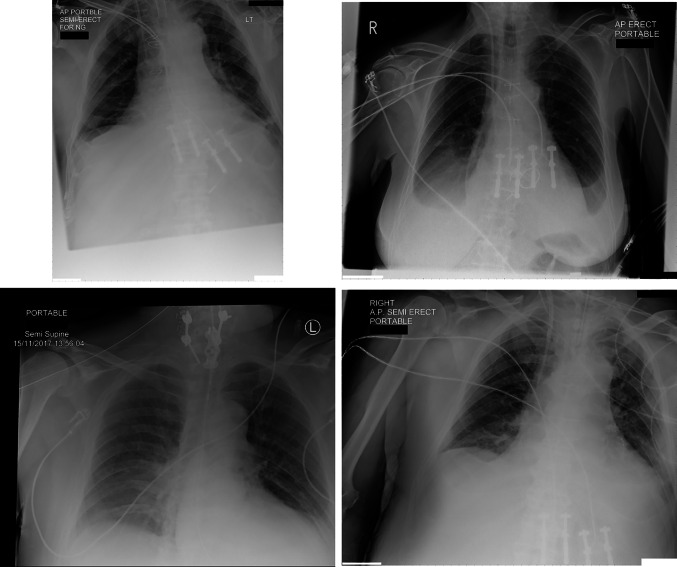
False positives across the whole data set: lines, tubes, and metalwork resulted in a small number of errors

**Fig. 5:**
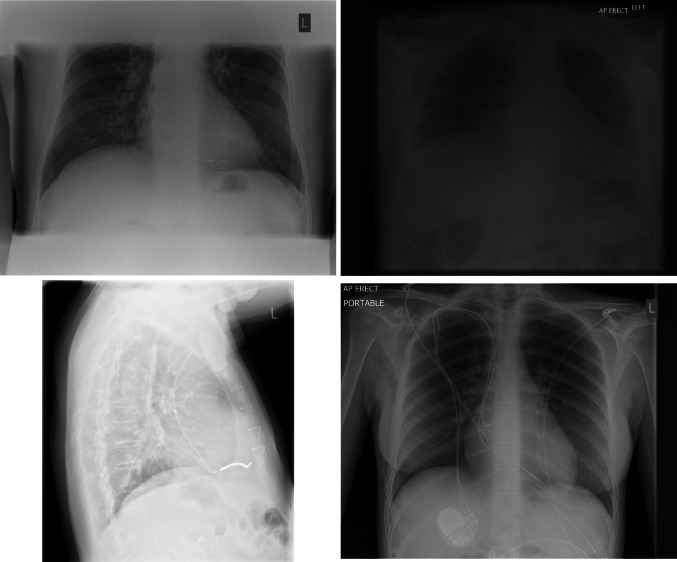
False negatives across the whole data set: poor contrast resolution, device box not included in image, and unusual orientation of the device resulted in a small number of errors

The misclassified false positive images were, unsurprisingly, composed of metallic artifact (electrocardiogram transponders and drains). False negative classification was associated with inability to see the pacemaker box, boxes positioned at the edge of the film, or only the wires present on the image (Fig. 4) and (Fig. 5).

## Discussion

Given a diagnostic quality chest radiograph, the model is excellent at picking up pacemakers, when present. Accuracy, although very high, is not 100%. For patient safety applications, this level of precision would not be suitable to replace current safety processes (even if a recent chest radiograph was available for all patients undergoing MRI). However, the computational resources required to run the model are low with few disadvantages.

The false positive results, although small, would create additional work for a human operator. We used a 50:50 split between positive and negative examples, which does not reflect the prevalence of pacemakers in the typical MRI patient population. Given the real world class-split, an anomaly detection model may be worth of future investigation.

Because the model accuracy was far higher than expected when designing the protocol and specifying the study size, the small test set was not sufficiently powered to analyze common patterns of model weakness. Repeating the project with more data and, specifically, a larger hold back test set, would enable improved model optimization on incorrectly classified examples (edge cases). With a large enough datasets, an ensemble model could allow screening for quality of image before checking the pacemaker.

Accuracy has not been formally assessed on cardiac device subgroups, for example implantable cardioverter defibrillators. Devices are continuously evolving; for example, leadless devices such as the *Medtronic Micra* device were not included in the data set. These have significantly different appearances on chest radiograph; no assessment of how the classification model would behave in these cases has been made. Abandoned leads may not be reliably identified by this model as these made up a minority of the training examples. As devices change and problems with the original model emerge, it is a challenge to make small adjustments to the model without retraining from scratch. The search could have been formulated to be more inclusive of other devices to build a model with proven performance at recognition of other devices such as AICDs.

The authors feel these points may illustrate some gap between expectations of artificial intelligence techniques and real-world performance in the safety–critical healthcare environment. In many cases, fixing errors and improving models without retraining from scratch would require considerable additional work. The machine learning model does not understand the cause behind the result and cannot be retrained based on underlying concepts [23].

For any specific question, building networks highly focused on a single question with curated, high quality datasets is likely to result in the best performance. For this reason, the model demonstrated excellent performance. There were a few incorrectly classified examples, reflecting unanticipated consequences of data set collection technique.

We chose to include all radiographs that had been selected from the sequential search. This had unintended consequences: for example, more lateral chest radiographs were included in the paced set, as lateral images are frequently done after a pacemaker is inserted. This information leak resulted in a final model more likely to predict a pacemaker on lateral projection. An ensemble of neural networks could be used to check the suitability of the input, mitigating this problem.

Pacemaker presence is a relatively simple problem, in most cases, very easily solved by a human and with no perceptual subjectivity. Despite best efforts to create a robust model, systemic weaknesses were easily identified but only after data curation and model assessment. Additional improvement could be realized by re-collecting and re-curating (a relatively expensive process). Alternatively, additional neural networks could be used in a pipeline (an ensemble of neural networks) to check the quality of a radiograph before analysis.

In addition to improved accuracy, further work could look at reliable identification of the brand of pacemaker and leads to aid MRI safety. Older (pre-2011) legacy devices are invariably MRI unsafe so precise device characterization can be useful. Labels or symbols on devices can aid identification, in some cases, but an image recognition tool may provide additional reassurance. Work from another institution has looked at this previously but with a relatively small number of samples [24].

There is much focus on using artificial intelligence for guiding diagnosis [25]. However, there are many possible applications of computer vision techniques for optimizing workflow and safety. In this study, we have demonstrated the potential for an artificial intelligence model to detect pacemakers on routine chest radiographs. This could be incorporated into current MRI safety processes to improve early identification, before safety questionnaire data is available.

## Conclusion

An InceptionV3-based neural network achieved very high accuracy for this image classification application. This would be a very useful addition to current processes, enabling automatic screening for devices in advance of MRI appointment to provide additional assurance and book safety checks in advance.

A novel database search technique can reduce the expense of producing good quality training datasets. Creative search methodology can help improve the baseline data quality but human review is still essential for a production-grade model.

Future work with improved search methodology could include search terms for other devices including AICDs and leadless designs. Collecting more information on pacemaker types from cardiology data sources could allow construction of an advanced model that could perform accurate multi-class device classification.

## References

[1] Cunqueiro A, Lipton ML, Dym RJ, Jain VR, Sterman J, Scheinfeld MH (2019). Performing MRI on patients with MRI-conditional and non-conditional cardiac implantable electronic devices: an update for radiologists. Clin Radiol..

[2] Russo RJ, Costa HS, Silva PD, Anderson JL, Arshad A, Biederman RWW (2017). Assessing the Risks Associated with MRI in Patients with a Pacemaker or Defibrillator. N Engl J Med..

[3] Shinbane JS, Colletti PM, Shellock FG (2011). Magnetic resonance imaging in patients with cardiac pacemakers: era of ‘MR Conditional’ designs. J Cardiovasc Magn Reson..

[4] Kocyigit D, Abozeed M, Kwon DH, Flamm SD, Wilkoff BL, Jellis CL (2021). Predictors of Cardiac Implantable Electronic Device Artifact on Cardiac MRI: The Utility of a Device Related Score. Heart Lung Circ..

[5] Kalin R, Stanton MS (2005). Current Clinical Issues for MRI Scanning of Pacemaker and Defibrillator Patients. Pacing Clin Electrophysiol..

[6] Shellock FG, Spinazzi A (2008). MRI Safety Update 2008: Part 2, Screening Patients for MRI. Am J Roentgenol..

[7] Brady AP (2017). Error and discrepancy in radiology: inevitable or avoidable?. Insights Imaging..

[8] Syed M. Black box thinking: the surprising truth about success (and why some people never learn from their mistakes) [Internet]. 2015 [cited 2021 Jun 7]. Available from: https://www.overdrive.com/search?q=3D424063-F4A2-422B-B5A6-1DF22253BB0D

[9] Turing AM. I.—COMPUTING MACHINERY AND INTELLIGENCE. Mind. 1950 Oct 1;LIX(236):433–60.

[10] Chollet F (2018). Deep learning with Python.

[11] Kim DH, Wit H, Thurston M (2018). Artificial intelligence in the diagnosis of Parkinson’s disease from ioflupane-123 single-photon emission computed tomography dopamine transporter scans using transfer learning. Nucl Med Commun..

[12] Kim DH, MacKinnon T (2018). Artificial intelligence in fracture detection: transfer learning from deep convolutional neural networks. Clin Radiol..

[13] Kim D, Wit H, Thurston M, Long M, Maskell G, Strugnell M (2021). An artificial intelligence deep learning model for identification of small bowel obstruction on plain abdominal radiographs. Br J Radiol..

[14] Irvin J, Rajpurkar P, Ko M, Yu Y, Ciurea-Ilcus S, Chute C, et al. CheXpert: A Large Chest Radiograph Dataset with Uncertainty Labels and Expert Comparison. ArXiv190107031 Cs Eess [Internet]. 2019 Jan 21 [cited 2021 Jun 7]; Available from: http://arxiv.org/abs/1901.07031

[15] Sorkhabi MM, Saadat Khajeh M (2019). Classification of alveolar bone density using 3-D deep convolutional neural network in the cone-beam CT images: A 6-month clinical study. Measurement..

[16] Nelson A, Herron D, Rees G, Nachev P (2019). Predicting scheduled hospital attendance with artificial intelligence. Npj Digit Med..

[17] Eichelberg M, Riesmeier J, Wilkens T, Hewett AJ, Barth A, Jensch P. Ten years of medical imaging standardization and prototypical implementation: the DICOM standard and the OFFIS DICOM toolkit (DCMTK). In: Ratib OM, Huang HK, editors. San Diego, CA; 2004 [cited 2021 Jun 7]. p. 57. Available from: https://www.proceedings.spiedigitallibrary.org/proceeding.aspx?doi=10.1117/12.534853

[18] Chollet F, others. Keras [Internet]. 2015. Available from: https://keras.io

[19] Martín Abadi, Ashish Agarwal, Paul Barham, Eugene Brevdo, Zhifeng Chen, Craig Citro, et al. TensorFlow: Large-Scale Machine Learning on Heterogeneous Systems [Internet]. 2015. Available from: https://www.tensorflow.org/

[20] Kluyver T, Ragan-Kelley B, Pérez F, Granger B, Bussonnier M, Frederic J, Loizides F, Schmidt B (2016). Jupyter Notebooks – a publishing format for reproducible computational workflows. Positioning and Power in Academic Publishing: Players, Agents and Agendas.

[21] Simonyan K, Zisserman A. Very Deep Convolutional Networks for Large-Scale Image Recognition. ArXiv14091556 Cs [Internet]. 2015 Apr 10 [cited 2021 Apr 29]; Available from: http://arxiv.org/abs/1409.1556

[22] Szegedy C, Vanhoucke V, Ioffe S, Shlens J, Wojna Z. Rethinking the Inception Architecture for Computer Vision. ArXiv151200567 Cs [Internet]. 2015 Dec 11 [cited 2021 Jul 21]; Available from: http://arxiv.org/abs/1512.00567

[23] Pearl J, Mackenzie D (2018). The book of why: the new science of cause and effect.

[24] Howard JP, Fisher L, Shun-Shin MJ, Keene D, Arnold AD, Ahmad Y (2019). Cardiac Rhythm Device Identification Using Neural Networks. JACC Clin Electrophysiol..

[25] Esteva A, Chou K, Yeung S, Naik N, Madani A, Mottaghi A (2021). Deep learning-enabled medical computer vision. Npj Digit Med..

